# Mission Sequence Model and Deep Reinforcement Learning-Based Replanning Method for Multi-Satellite Observation

**DOI:** 10.3390/s25061707

**Published:** 2025-03-10

**Authors:** Peiyan Li, Peixing Cui, Huiquan Wang

**Affiliations:** School of Aeronautics and Astronautics, Zhejiang University, Hangzhou 310027, China; lipeiyan@zju.edu.cn (P.L.); cpx@zju.edu.cn (P.C.)

**Keywords:** Agile Earth Observation Satellites (AEOSs), mission replanning, deep reinforcement learning, attention mechanism, mission sequence model

## Abstract

With the rapid increase in the number of Earth Observation Satellites (EOSs), research on autonomous mission scheduling has become increasingly critical for optimizing satellite sensor operations. While most existing studies focus on static environments or initial planning states, few address the challenge of dynamic request replanning for real-time sensor management. In this paper, we tackle the problem of multi-satellite rapid mission replanning under dynamic batch-arrival observation requests. The objective is to maximize overall observation revenue while minimizing disruptions to the original scheme. We propose a framework that integrates stochastic master-satellite mission allocation with single-satellite replanning, supported by reactive scheduling policies trained via deep reinforcement learning. Our approach leverages mission sequence modeling with attention mechanisms and time-attitude-aware rotary positional encoding to guide replanning. Additionally, scalable embeddings are employed to handle varying volumes of dynamic requests. The mission allocation phase efficiently generates assignment solutions using a pointer network, while the replanning phase introduces a hybrid action space for direct task insertion. Both phases are formulated as Markov Decision Processes (MDPs) and optimized using the PPO algorithm. Extensive simulations demonstrate that our method significantly outperforms state-of-the-art approaches, achieving a 15.27% higher request insertion revenue rate and a 3.05% improvement in overall mission revenue rate, while maintaining a 1.17% lower modification rate and achieving faster computational speeds. This demonstrates the effectiveness of our approach in real-world satellite sensor applications.

## 1. Introduction

The past few decades have seen a rapid increase in Earth Observation Satellites (EOSs) in orbit. With advancements in on-board computation capabilities, communication systems, and attitude maneuvering abilities, EOSs have become increasingly important in various societal fields, such as environmental monitoring, public safety, and disaster prevention and response [1,2].

The essence of satellite observation scheduling lies in matching tasks to satellites and determining the precise observation time for each task [3]. Most existing studies focus on static scheduling scenarios, where all observation tasks are assumed to be predefined at once, and the scheduling results remain fixed throughout the process [4]. The diverse demands for Earth observation and the complexity of observation tasks have led to the need for efficient satellite scheduling [5]. Most current studies focus on static scheduling scenarios, where all observation tasks are assumed to be deterministic and remain fixed throughout the scheduling process. However, in real-world satellite mission planning scenarios, there are both internal uncertainties (e.g., partial satellite equipment failures) and external uncertainties (e.g., changes in observation opportunities or user demand), which make the planning process highly dynamic [6]. These factors lead to the episodic and unpredictable nature of observation requests, highlighting the need for continuous adjustment and adaptation of mission replanning in response to unexpected emergencies [7].

Dynamic requests are typically delivered in batches with uncertain scales, requiring coordinated efforts among multiple satellites to meet rescheduling demands. In urgent scenarios, an efficient planning mechanism is essential to reduce problem complexity and alleviate the computational burden on satellites [8]. For dynamic scheduling in emergency tasks, many studies suggest that it is important to insert new tasks while minimizing changes to the original plan, which can benefit subsequent routine operations [9]. Minimizing disturbances to the original planning scheme is significant for three main reasons: First, it ensures the stability and continuity of the satellite observation system [10], which is especially important when task arrivals become regular. Without this consideration, the planning scheme may require frequent revisions [11]. Second, for periodic observation targets, such as tracking or astronomical observation tasks, replanning may undermine the beneficial features of the original scheme. Tasks from the original plan could be removed, making it difficult to stably meet user requirements and leading to resource wastage [12]. Lastly, due to the characteristics of Agile Earth Observation Satellites (AEOSs), the execution time of observation tasks is closely linked to imaging quality. Even minor perturbations to the original plan could affect the actual observation outcomes [8].

The operations of AEOSs inherently involve time-dependent mission sequences due to attitude transitions and the associated resource consumption between observations. To improve the quality of replanning solutions, it is essential to ensure maneuver continuity and efficiency [13]. This requires accurate modeling and real-time awareness of satellite scheme states, effectively integrated with rapid task replanning.

With the rise of onboard computing capabilities, autonomous satellite scheduling is becoming a key trend, enabling rapid responses to dynamic requests while reducing reliance on ground control [14]. However, existing static scheduling algorithms often suffer from high computational overhead and poor performance when handling batch arriving requests. Recently, deep reinforcement learning (DRL) has been widely adopted for solving combinatorial optimization problems [15], demonstrating advantages in fast inference and strong generalization. We believe that well-trained, end-to-end inference models are well suited to address the challenge of efficiently responding to large-scale observation requests.

Therefore, the main goal of this study is to investigate the mission replanning problem for multiple AEOS on dynamic arrival of observation requests. We propose a mission sequence-based replanning (MSRP) algorithm tailored for multi-satellite systems. MSRP introduces a temporary master–slave architecture for task allocation and single-satellite-level replanning. By a customized attention mechanism, the algorithm uses time-attitude information to enhance the modeling of sequence dependencies. Mission allocation and replanning decisions are made sequentially in two stages based on satellite states, with both phases jointly optimized to ensure high-quality strategy generation.

The contributions of this paper are as follows.

1. To the best of our knowledge, this is the first work to directly model satellite observation sequences using deep learning, incorporating time and attitude information. We utilize task timing and side-swing angles as positional encodings for self-attention modeling and introduce a gated global pooling method based on interval information to generate a comprehensive satellite state representation. Ablation studies and comparisons with classical methods demonstrate that our approach significantly improves mission replanning performance.
2. We propose a DRL-based mission replanning algorithm for a single satellite, designed to handle discrete-continuous hybrid decision variables. This algorithm achieves dual-objective optimization by maximizing task revenues and minimizing changes to the original plan. Experimental results validate the effectiveness of the algorithm, demonstrating its generalization ability across different-scale scenarios.
3. We propose an on-site mission allocation algorithm for request-receiving satellites, integrating cross-attention mechanisms and pointer networks to provide end-to-end allocation solutions. Combined with the replanning method, this two-stage optimization approach demonstrates the advantages of our algorithm over classical methods.

The rest of this article is organized as follows. Section 2 provides a review of previous research on satellite task replanning techniques. Section 3 presents our solution framework in the scenario, along with the optimization objectives and constraints of the problem. Section 4 introduces the mission sequence modeling and dynamic request modeling approach, along with the DRL-based mission allocation and replanning algorithms. In Section 5, a series of experiments and analyses are conducted to verify the effectiveness of the proposed methods. Finally, Section 6 concludes the paper.

## 2. Literature Review

### 2.1. Dynamic Scheduling of AEOSs

An increasing amount of research focuses on the dynamic scheduling of Agile Earth Observation Satellites (AEOSs). Pemberton and Greenwald [16] described the dynamic scheduling of EOS and analyzed the various emergency conditions associated with it. Liang et al. [17] developed a scalable modeling method based on predicate logic and proposed a knowledge-based, priority rule-driven heuristic approach for rapid responses. Wen et al. [18] introduced a multi-scenario fusion-based onboard scheduling and coordination approach, which transforms complex onboard rescheduling problems into a solution selection problem from ground-generated solutions. He et al. [13] proposed a hierarchical scheduling method for real-time scheduling, which dynamically adjusts scheduling precision based on task observation times, thereby improving the overall algorithm efficiency. Han et al. [19] utilized a chance-constrained programming model to describe uncertainty and introduced an improved simulated annealing-based heuristic, along with a fast insertion strategy for large-scale observation tasks. Li et al. [14] proposed a low-complexity onboard periodic rescheduling algorithm based on a rolling horizon optimization strategy, integrating greedy-based allocation algorithms, pointer network-based scheduling algorithms, and local iterative search algorithms to provide high-quality solutions. Liu et al. [20] combined task planning with transient scheduling, proposing a simultaneous planning and execution strategy along with a heuristic rolling schedule to address dynamic scheduling without considering initial plans.

In emergency situations, various task information must be comprehensively considered, including users’ expected completion times, time windows, inter-task intervals, and dynamic environmental factors, adding more complexity to task scheduling [8]. Wei et al. [21] considered the transient transition time between requests and proposed a multi-objective memetic approach that accounts for time-dependent transition times. Peng et al. [22] modeled the dependency of transition times between consecutive observations and developed a greedy random iterative local search method for fast feasibility checks and task allocation. Du et al. [23] employed a task clustering preprocessing method to enhance task scheduling efficiency by combining potential objectives. Du et al. [24] trained a probability prediction model using historical satellite scheduling data to improve task allocation strategies. Song et al. [25] proposed a graph-structured mixed-integer mathematical model, introducing satellite scheduling knowledge to develop a genetic algorithm, which experimentally demonstrated its effectiveness in improving system operation efficiency.

However, in the research on the rescheduling of observation tasks, few studies directly model the original task sequence. These studies often overlook the influence of original task execution time and resource demands on the dynamic insertion of new requests.

### 2.2. Multi-Satellite Scheduling Architecture

Multi-satellite cooperative autonomous mission planning can typically be decomposed into a top-level multi-satellite task allocation problem and a bottom-level single-satellite task scheduling problem [26,27]. Bianchessi et al. [28] proposed a three-phase scheduling method to reduce the problem’s complexity, which includes request analysis, task allocation, and a distributed optimization approach. Li et al. [29] constructed a dedicated experimental platform for coordinating tasks between geostationary and low Earth orbit satellites and proposed a task allocation algorithm tailored for this platform. Qi et al. [30] designed an evolutionary ant colony optimization method and an interactive rescheduling approach for the task pre-planning and re-planning phases, respectively, to maximize observation profit and achieve load balancing. Liu et al. [31] addressed the single-satellite scheduling problem based on Q-networks and introduced a profit-based competitive strategy to resolve scheduling conflicts in multi-satellite systems, thereby reducing the burden on cooperative communications. Most of the studies above focus on static tasks, with few designs addressing task rescheduling. Du et al. [23] proposed a new multi-dimensional multi-agent collaborative model and a secondary allocation strategy based on contract network protocols (CNPs) to reduce task allocation conflicts and efficiently minimize losses in case of failure by reinserting tasks. Chen et al. [32] introduced a hierarchical task planning framework, where the global planner divides the clusters, and the local planner searches for the optimal solution using Discrete Particle Swarm Optimization (DPSO) algorithms. In emergency situations, task priorities are used to reschedule part of the cluster tasks.

Some multi-agent system (MAS) optimization algorithms have been applied to satellite task allocation. Yang et al. [33] proposed a dynamic distributed architecture using an improved contract net protocol and blackboard model for task coordination but did not address constraints on revising original plans. Li et al. [34] introduced an iterative coordination model based on Nash Equilibrium (NE) theory to solve joint space observation problems but overlooked task allocation under emergency conditions. MAS-based methods often require multiple coordination rounds, limiting their suitability for rapid-response scenarios.

Recent advancements in multi-agent reinforcement learning (MARL) have inspired new approaches to dynamic task allocation. Many studies on dynamic task coordination focus on unmanned aerial vehicles (UAVs). For example, Liu et al. [35] addressed dynamic task allocation for heterogeneous UAVs by proposing a proposer-responder mechanism and implementing a Q-network for response generation. This mechanism effectively models the dynamic process of task request arrival, allocation, and processing.

In satellite observation mission allocation, Wang et al. [9] applied transfer learning to adapt historical strategies into current initial strategies and developed a hybrid MARL algorithm for dynamic task planning. This approach focuses on action learning but lacks knowledge transfer, limiting its effectiveness with heterogeneous agents. Li et al. [36] replaced the contract net protocol with a multi-agent deep deterministic policy gradient (MADDPG) algorithm to solve real-time multi-satellite cooperative observation scheduling, reducing communication overhead. Saeed et al. [37] proposed a multi-agent, multi-target reinforcement learning framework for dynamic task and sensor resource matching but did not address satellite maneuvering constraints. Zhang et al. [38] used the multi-agent proximal policy optimization (MAPPO) algorithm for task scheduling under satellite state constraints. However, it does not adapt to dynamic changes in satellite resources, environmental conditions, or observation tasks. Essentially, it offers a rapid solution for static scenarios.

In summary, most MARL-based satellite mission planning methods focus on solving the initial allocation problem, with limited attention to the replanning problem. Existing approaches rely on fast neural network inference to handle dynamic situations but lack comprehensive modeling of the environment state. Additionally, this paper focuses on task allocation by individual satellites based on received requests, where each satellite directly assigns tasks according to its strategy. The issue of collaborative allocation, as typically explored in MARL research, is not considered in this study.

### 2.3. General Models and Algorithms

The scheduling problem of multiple Earth Observation Satellites is a complex combinatorial optimization problem and has been proven to be NP-hard [39].

Task planning models for multi-satellite observation missions include integer programming models [40], constraint satisfaction models [41], and graph-based models [42]. The classical solution algorithms include exact algorithms [43,44], heuristic methods [45,46] and meta-heuristics methods [47]. For large-scale problems, constructing an exact model is practically impossible. Heuristic or metaheuristic methods can be used to optimize the search strategy for solving the problem. Li et al. [48] designed a genetic-evolutionary bi-level solution framework, using an improved genetic algorithm to separately solve the task allocation and resource scheduling problems. Yang et al. proposed a hybrid local replanning strategy for multi-satellite imaging mission planning in uncertain environments, enhancing the adaptive differential evolution algorithm. This method integrates effective task insertion rules based on task observation order [7]. Zheng et al. [49] employed a multi-objective hybrid dynamic mutation genetic algorithm combined with periodic and near-real-time replanning techniques for onboard replanning in disruptive scenarios.

These methods rely on specifically designed heuristic strategies, may lead to local optima [50], and are unable to meet the real-time computation requirements of large-scale dynamic scenarios. Recently, due to the powerful modeling and reasoning capabilities of neural networks, they have also been applied to problem-solving.

Neural networks are used as predictive models to assist in real-time decision making. Chen et al. [51] introduced a real-time multi-satellite scheduling approach that combines a hierarchical prediction model based on a stacked multi-channel transformer network with a heuristic local search algorithm, achieving high-quality initial solutions and rapid computation. Gu et al. [52] enhanced the replanning algorithm’s effectiveness by utilizing satellite cloud image forecasting information from predictive recurrent neural networks.

Another approach is to directly use neural networks to build decision-making strategies. Deep reinforcement learning (DRL) continuously learns and optimizes scheduling strategies through feedback from the environment, enabling it to make optimal decisions based on relevant states. This method has shown great potential in solving multiple AEOS scheduling problems. For instance, Wang et al. [53] developed a satellite scheduling model based on the dynamic stochastic knapsack problem and optimized it using a deep reinforcement learning framework to maximize total expected profit, achieving real-time scheduling for imaging satellites. However, this model focuses solely on immediate request responses, neglecting the updates to long-term mission plans.

Many graph neural network (GNN) models have been used to enhance the representation of task planning problems [54]. Wang et al. [55] modeled the multi-satellite scheduling problem as a heterogeneous graph subject to various constraints, using a GNN and Transformer based encoder to enhance information representation. They trained a two-stage dynamic decoder using reinforcement learning. Feng et al. [56] utilized GNNs to extract feature knowledge of large-scale tasks and their constraint relationships while employing a metaheuristic optimization module to resolve dynamic constraint conflicts.

However, graph-based models often represent edges as various constraints to avoid conflicts, which is not suitable for replanning scenarios. This modeling approach struggles to capture the original sequence and handle task replacement, potentially leading to inflexibility in task insertion. Additionally, for dynamic requests arrival and insertion in our study, GNNs require frequent updates to the graph structure, resulting in significant computational overhead.

The sequence-to-sequence (seq2seq) model is an advanced combinatorial optimization framework that provides an encode-decode strategy learning paradigm. Pointer networks (Ptr-Net) [57] and Transformer architectures [58] have been proven effective in solving problems like the vehicle routing problem (VRP) and traveling salesman problem (TSP). Liu et al. [59] proposed a model with a local attention mechanism to reduce the search space in the autoregressive process, improving decision quality. Chen et al. [60] used attention layers to model dynamic time dependencies between satellite tasks, training an encoder–decoder neural network with the REINFORCE algorithm. Li et al. [14] used a trained pointer network to generate single EOS scheduling solutions in an end-to-end manner for rapid onboard replanning. Liang et al. [61] implemented rapid rescheduling using an improved pointer network with a masking mechanism to maximize the completion profit of dynamic tasks while minimizing the impact on scheduled tasks. Long et al. [62] proposed a scalable task planning method based on a Transformer model with time encoding, which we consider as one of the baselines in this study.

Multi-satellite task allocation involves specific constraints and is a structured sequential decision-making process. The attention-based Ptr-Net uses a softmax probability distribution as a pointer of the input sequence to gradually select input elements to construct the output sequence, making it effective for handling long sequences and combinatorial optimization problems of varying scales. Particularly for replanning problems, where the number of incoming requests and satellites is uncertain, our method employs a dynamic encoder and a pointer network-based decoder to handle dynamic task allocation. Compared to the Transformer model, the pointer network has a lower computational complexity, directly outputs the strategy probability distribution, and is better suited for discrete decision problems like task allocation.

## 3. Problem Statement

This section first introduces the scenario and the replanning framework, followed by a description of the definitions and assumptions. Next, the objective function and constraints of the problem are presented.

### 3.1. Scenario and Replanning Framework

**Figure 1:**
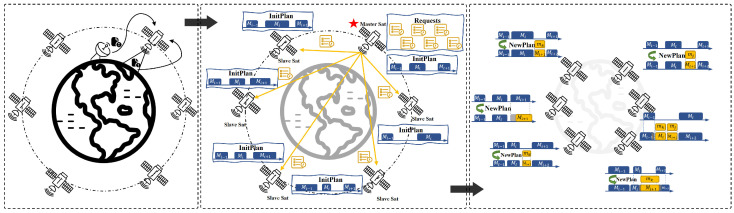
Scenario and framework of multi-satellite mission replanning.

During orbital operations, satellites may encounter various environmental changes that could render original observation tasks invalid or require modifications. Internal factors include satellite payload or platform failures that lead to task cancellations, which in our scenario are handled as dynamic requests directed to the nearest available satellite. External factors include changes in observation conditions, such as cloud cover or lighting, which may result in task cancellations or suspensions, as well as shifts in user demand or emergency observation requests triggered by natural disasters. As shown in Figure 1, dynamic requests originate from ground user demands, autonomous satellite discoveries, or unforeseen events. We assume that these requests initially reach individual satellite nodes.

For scenarios involving batch arrivals of dynamic observation requests and their timely scheduling, we propose a framework combined with mission allocation and single-satellite replanning. Based on the assumption of a distributed constellation where each node has equal intelligence, we adopt a temporary master–slave architecture to minimize the demands of communication resources for mission allocation. Specifically, the satellite that first receives the requests acts as the temporary master, while other satellites serve as slaves.

By modeling the original plans of each satellite, the master satellite can directly generate high-quality allocation schemes for slave satellites. Once the allocation is received, each satellite executes its own single-satellite replanning. This approach reduces the overall scale of problem solving, minimizes communication and negotiation overhead, and provides precise replanning solutions tailored to the actual state of each satellite. We believe that it achieves more accurate responses while minimizing modifications to the original plans.

### 3.2. Assumptions and Definitions

Our scenario can be described as follows.

1. Assuming that a group of satellites $Sat=\{sat_{j}|j=1,2,\ldots ,N_{S}\}$. Each satellite is represented as $sat_{j}=\left\{v^{j},a_{max}^{j},C_{mem}^{j},C_{power}^{j},a^{j},h_{mem}^{j},h_{power}^{j}\right\}$, where $v^{j},a_{max}^{j},C_{mem}^{j},C_{power}^{j}$ are static parameters that remain constant during the scheduling process. These parameters, which reflect inherent task characteristics, are pre-determined and include the attitude transition rate ($v^{j}$), maximum side swing angle ($a_{max}^{j}$), memory capacity ($C_{mem}^{j}$), and power capacity ($C_{power}^{j}$). Conversely, $a^{j},h_{mem}^{j},h_{power}^{j}$ are dynamic parameters, which are updated at each scheduling step to represent the current side swing angle ($a^{j}$), remaining memory ($h_{mem}^{j}$), and remaining power ($h_{power}^{j}$) at any given time. It is important to note that, in our model, $sat_{j}$ operates continuously on orbit $Orbit^{j}$ and does not perform any orbit-raising maneuvers.
2. Dynamic requests can be represented as $Req=\{m_{i}|i=1,2,\ldots ,N_{R}\}$. Each request is defined as $m_{i}=\left\{\left(s_{i}^{j},e_{i}^{j},d_{i}^{j},a_{i}^{j},p_{i},mem_{i},power_{i}\right)|j=1,2,\ldots ,N_{S}\right\}$. It contains a quadruple, $s_{i}^{j},e_{i}^{j}$ are the start time and end time of the visible time window of $sat_{j}$ to request $m_{i}$; $d_{i}^{j}$ is the execution duration required for $sat_{j}$; $a_{i}^{j}$ represents the side swing angle required for $sat_{j}$ observation to $m_{i}$. Additionally, the request includes the priority $p_{i}$, required memory consumption ${mem}_{i}$, and required power consumption ${power}_{i}$.
3. When a request is scheduled into the mission sequence of a designated satellite $sat_{j}$, it is represented as $M_{i}=\left\{m_{k},r_{k},\left(Ts_{i},Te_{i}\right),sat_{j}\right\}$, where $\left(Ts_{i},Te_{i}\right)$ represent the start and end time of the mission execution, and $r_{k}$ indicates the reward associated with the task. Each satellite has an optimal initial plan $InitPlan_{j}=\left\{M_{1}^{j}\to M_{2}^{j}\to \ldots \to M_{L_{j}}^{j}\right\}$, that contains $L_{j}$ missions; The updated mission plan is represented as $NewPlan_{j}=\left\{M_{1}^{j}\to M_{2}^{j}\to \ldots \to M_{L_{j}^{\prime }}^{j}\right\}$, with a length of $L_{j}^{\prime }$.
4. We define that new requests arrive in batches, and at each time step, only one satellite will be designated as the master satellite for task allocation.
5. The integration of new requests into the mission sequence must satisfy the constraints of the satellite imaging tasks.

### 3.3. Objective Function and Constraints

The objective function in this work consists of two main components: mission revenues and disturbance costs. We define mission priority as its revenue, aiming to maximize the total revenue generated by the satellite from executing the target observation in one episode. Disturbance costs represent the impact of inserting new tasks into the initial plan throughout the replanning process, including time changes and task substitutions. The objective function we design is as follows:

(1) $$maxf=\mu \cdot f_{1}-\left(1-\mu \right)\cdot f_{2}$$

(2) $$f_{1}=\sum_{j}^{N_{S}}\sum_{i}^{L_{j}^{\prime }}p_{i},f_{2}=\sum_{j}^{N_{S}}\sum_{i}^{L_{j}}p_{i}\cdot \tau_{change}$$

(3) $$\tau_{change}=\left\{\begin{array}{ccccccccc} \omega_{1}, & \; & if & \; & m_{i} & \; & is & \; & removed,\\ \omega_{2}, & \; & if & \; & m_{i} & \; & is & \; & shifted,\\ 0, & \; & if & \; & m_{i} & \; & is & \; & unchanged, \end{array}\right.$$

where μ is the scale scaling factor, which can be adjusted based on the user preferences. $f_{1}$ represents the total revenue of the new plan, and $f_{2}$ is the penalty for changes to the initial plan. The changes are classified into three categories, each assigned a different penalty factor $0<\omega_{2}<\omega_{1}\leq 1$: removed, execution time shifted, and no change occurred.

The following constraints should be satisfied.

1. Visible Time Window Constraint: The start and end of the execution time in the satellite’s mission sequence must satisfy
  (4) $$\left\{\begin{array}{c} Ts_{i}^{j}\geq s_{i}^{j},\\ Te_{i}^{j}=Ts_{i}^{j}+d_{i}^{j}\leq e_{i}^{j}, \end{array}\right.$$
2. Transition Time Constraint: Continuous missions must satisfy the attitude transition requirement. After completion of the previous task $M_{i}$, the satellite must undergo a specified maneuver, ensuring that $M_{i+1}$ can still complete the observation before the end of its visible time window.
  (5) $$Te_{i}+\frac{\left|a_{i}-a_{i+1}\right|}{v}+d_{i+1}\leq e_{i+1}$$
3. Adjustment Time Range Constraint: The time window within which $M_{i}$ can be moved is determined by its visible time window $\left(s_{i},e_{i}\right)$, as well as the execution times of the preceding task $M_{i-1}$ and the following task $M_{i+1}$. The maximum advancement time is
  (6) $$T_{forward}=Ts_{i}-max\left\{s_{i},Te_{i-1}+\frac{\left|a_{i}-a_{i-1}\right|}{v}\right\}$$
  The maximum delay time is
  (7) $$T_{backward}=min\left\{e_{i},Ts_{i+1}-\frac{\left|a_{i+1}-a_{i}\right|}{v}\right\}-Te_{i}$$
4. Uniqueness constraints: Each request has a different visible time window for each satellite, but it can only be executed by one satellite at a time.
  (8) $$NewPlan_{j}\cap {NewPlan}_{k}=\emptyset ,\forall j,k=1,2,\ldots ,N_{S}$$
  (9) $$InitPlan_{j}\cap {InitPlan}_{k}=\emptyset ,\forall j,k=1,2,\ldots ,N_{S}$$
  Each task can only be observed once. For any given satellite, each selected task can have at most one preceding task and one succeeding task.
5. Resource Constraint: The complete $NewPlan_{j}$ is managed through the removal of resource-exceeding tasks from its terminal end.
  (10) $$\sum_{i}^{L_{j}^{\prime }}{mem}_{i}\leq C_{mem}^{j},\forall j=1,2,\ldots ,N_{S}$$
  (11) $$\sum_{i}^{L_{j}^{\prime }}{power}_{i}\leq C_{power}^{j},\forall j=1,2,\ldots ,N_{S}$$

## 4. Solution Method

This section presents a mission sequence-based replanning (MSRP) algorithm for multi-satellite observation. To address the complexity of original plans and the uncertainty of request scales, we propose mission sequence modeling and dynamic request embedding to enhance state representation. As described in Section 3.1, the MSRP framework consists of two components. In mission allocation, we design a pointer network-based algorithm to generate assignment solutions. In the satellite replanning part, we introduce a neural network-based task insertion strategy to select tasks and determine execution times for received requests. Algorithm 1 outlines the MSRP framework. Both components are modeled as Markov Decision Processes (MDPs) and trained using the proximal policy optimization (PPO) algorithm.
**Algorithm 1** The framework of MSRP **Input:** Satellite set Sat, Request set Req arrive at $sat^{*}$, original plan $\left\{InitPlan_{j}|j=1,2,\ldots ,N_{S}\right\}$ **Output:** New plan $\left\{NewPlan_{j}|j=1,2,\ldots ,N_{S}\right\}$  Enhance State Representation: $E_{MS},E_{GS}=MissionSequenceModel\left(Sat,InitPlan\right)$ $E_{m},E_{R}=DynamicRequestModel\left(Req\right)$  Mission Allocation in $sat^{*}$: **for** $m_{i}\in Req$ **do**  Select $sat_{j}\in Sat$ through $APolicy(E_{GS},E_{R},m_{i})$  Assign $m_{i}$ to $Req_{j}$ **end for** **return** $Req_{j}$ for each $sat_{j}$  /*proceed in parallel*/ Mission Replanning in $sat_{j}$: **while** not terminated **do**  Get $\left(m_{i},Ts_{i}\right)$ though $RPolicy(E_{M},E_{m},sat_{j})$  Insert $m_{i}$ into $Newplan_{j}$ **end while**  Output Optimal Scheme $\left\{NewPlan_{j}\right\}$

### 4.1. Mission Sequence Modeling

**Figure 2:**
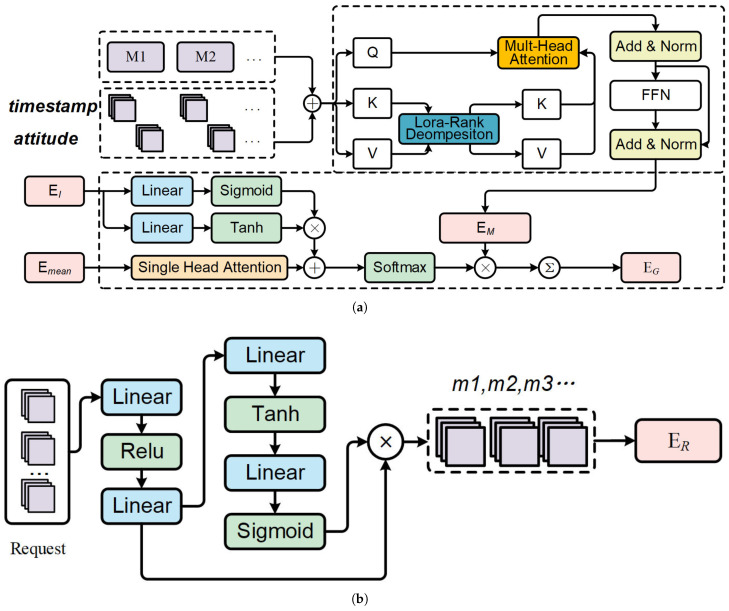
The structure of mission sequence modeling and dynamic request modeling method. (**a**) Mission sequence modeling. (**b**) Dynamic request modeling.

We introduce a spatiotemporal embedding method for modeling mission sequences, capturing both time and space-related factors. Temporal aspects focus on task execution times (start times and intervals), while spatial factors address the required observation side swing angles. By employing attention blocks, we encode both timestamp and maneuver information into embedding vectors. A gating mechanism integrates the mission intervals, followed by global pooling to produce a unified representation of the current satellite state. This approach effectively combines spatiotemporal dynamics for enhanced task sequence modeling. The complete structure of the method is fully displayed in Figure 2a.

To ensure the timeliness of request responses, we fix the length of the mission sequence to *L*. If the sequence length exceeds *L*, we truncate it to the *L* tasks closest to the decision time. If length is less than *L*, we append virtual missions at the end, with their execution time set to the orbit end time and revenues set to 0.

First, the input satellite planning schemes $InitPlan=\{M_{1}\to M_{2}\to \ldots \to M_{L}\}$ are transformed into high-dimensional representations by embedding each mission $M_{i}$ into a vector $e_{i}$. These individual embeddings are then concatenated to form the vector $E=\left[e_{1},e_{2},\ldots ,e_{L}\right]\in R^{L\times d}$, which represents the entire mission sequence in a continuous vector space.

**Figure 3:**
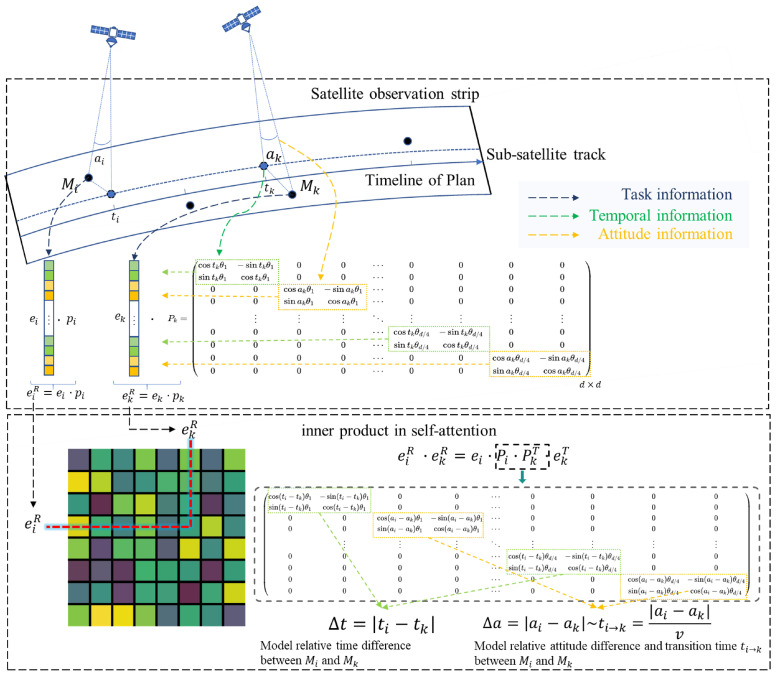
The schematic diagram of the rotational positional encoding mechanism.

Inspired by the multilevel temporal information rotational position encoding method in [63], we embed the timestamp information from the original missions and the side swing angles for request–satellite pairs, modeling the temporal correlations between mission sequence. By incorporating temporal and attitude information, we enhance the attention mechanism to explicitly capture the absolute timing information of the tasks and calculate the relative transition span between tasks. Figure 3 illustrates the schematic diagram of the embedding process.

We standardize the time granularity for all tasks using the midpoint of each execution time as the timestamp, expressed as $TimeStamps=\{t_{i}=\frac{st_{i}+et_{i}}{2},i=1,2,\ldots ,L\}$.

Satellites require attitude adjustments between consecutive observations. In our model, transmission time is proportional to the side swing angle difference between adjacent tasks. Therefore, we utilize $Attitude=\{a_{1},a_{2},\ldots ,a_{L}\}$ to effectively enhance the learning of the transition interval information between tasks.

For a mission vector $e_{i}\in E$, it encodes the timestamp and attitude positions into a *d*-dim space with the following function.

(12) $$e_{i}^{R}=e_{i}\cdot P_{i}$$

where $P_{i}=\left[P_{i}^{1}\;P_{i}^{2}\;\ldots \;P_{i}^{d/4}\right]\in R^{d\times d}$ is a position embedding matrix. Each sub-matrix $P_{i}^{j}\in R^{4\times 4}$ is

(13) $$P_{i}^{j}=\left[\begin{array}{cccc} cos(t_{i}\theta_{j}) & -sin(t_{i}\theta_{j}) & 0 & 0\\ sin(t_{i}\theta_{j}) & cos(t_{i}\theta_{j}) & 0 & 0\\ 0 & 0 & cos(a_{i}\theta_{j}) & -sin(a_{i}\theta_{j})\\ 0 & 0 & sin(a_{i}\theta_{j}) & cos(a_{i}\theta_{j}) \end{array}\right]$$

where $\left\{\theta_{j}=10000^{-4(j-1)/d},j=1,2,\ldots ,d/4\right\}$ are pre-defined. Due to the sparsity of $P_{i}$, the computation can be accelerated by performing element-wise multiplication and addition operations.

The modeling of time-attitude dependencies is achieved through the computation of self-attention mechanisms. As illustrated in Figure 3, the pairwise inner product calculations effectively capture the relative time differences and relative side swing angle variations between tasks. It is evident that the sinusoidal and cosine functions in the encoding enable $e_{k}^{R}$ to be represented as a function of $e_{i}^{R}$ for any given execution time and attitude offset Δt,Δa (as shown in Equation (14)), allowing the model to efficiently learn and focus on relative time-attitude differences.

(14) $$e_{i}^{R}\cdot e_{k}^{R}=e_{i}P_{i}P_{k}^{⊤}e_{k}^{⊤}=g\left(e_{i},e_{k},\Delta t_{i-k},\Delta a_{i-k}\right)$$

Moreover, integrating attitude information provides richer insights than relying solely on task execution times, as the relative side swing angle difference is proportional to the transition time between tasks: $t_{i\to i+1}=\frac{\left|a_{i+1}-a_{i}\right|}{v}$, enabling more precise modeling.

This encoding adapts to variations in visible time windows induced by different task distributions and satellite configurations. Unlike conventional positional encoding, it remains unaffected by task sequence order changes, making it more robust for replanning.

After adding rotational position encodings, we obtain the mission vector $E_{P}$, which then is sent to a multi-head self-attention block. First, $E_{P}$ is linearly mapped through query, key, and value components to obtain three feature matrices:

(15) $$Q,K,V=E_{P}W_{Q},E_{P}W_{K},E_{P}W_{V}$$

where $Q,K,V\in R^{n\times d}$ and $W_{Q,K,V}\in R^{d\times d}$. We add a low-rank decomposition projection to generate a more compact contextual representation. The corresponding K,V are reduced in size by a trainable mapping function $h:E\in R^{L\times d}\to D\in R^{l\times d}$. Here, *l* is an optional size, representing the range of selected correlations.

(16) $$D_{K}=h\left(K\right)={\left(Softmax\left(KW_{l}^{⊤}\right)\right)}^{⊤}K$$

(17) $$D_{V}=h\left(V\right)={\left(Softmax\left(VW_{l}^{⊤}\right)\right)}^{⊤}V$$

where $D_{K},D_{V}\in R^{l\times d}$ and $W_{l}\in R^{l\times d}$ is trainable.

Afterward, the input *Q* is passed through the attention mechanism to compute the embeddings.

(18) $$A=Softmax\left(\frac{QD_{K}^{⊤}}{\sqrt{d/h}}\right)D_{V}$$

The final output is $E_{M}\in R^{L\times d}$ which merged with multi-head and through linear layers. The embedding of each satellite is then concatenated and reshaped back into $E_{MS}\in R^{N_{S}\times L\times d}$.

In this module, we design a global pooling method for mission sequences to capture the satellite’s overall schedule. It combines a virtual node-based attention mechanism to weight $E_{M}$ and a gating mechanism that uses execution durations and task intervals. These are then combined and weighted-summed with $E_{M}$ to obtain each satellite’s state representation.

We use the average of the task embeddings $e_{mean}=\frac{1}{L}\sum_{i=1}^{L}E_{M}$ as the virtual node. Then, a single-head attention layer is utilized to calculate the weight $\alpha_{i}$ for each mission.

(19) $$\alpha_{i}=\frac{exp(e_{mean}e_{i}^{⊤})}{\sum_{k}exp\left(e_{mean}e_{k}^{⊤}\right)}$$

During observation schedule adjustments, requests may need to be inserted into or replace existing missions, taking into account the preceding and following intervals. Additionally, since task durations vary and cannot be treated as single time points, their execution times must also be considered.

In $Plan=\{M_{1}\to M_{2}\to \ldots \to M_{L}\}$, for each $M_{i}$, we consider a triplet $I_{i}=\left(I_{i}^{left},d_{i},I_{i}^{right}\right)$, where $I_{i}^{left}=Ts_{i}-Te_{i-1}$, $I_{i}^{right}=Ts_{i+1}-Te_{i}$, and $d_{i}$ is the execution duration of $M_{i}$.

We incorporate mission interval information $E_{I}$ by calculating intermediate features and assigning weights through a gating mechanism, allowing the model to identify important task intervals and adaptively fuse the corresponding features.

(20) $$E_{I}^{*}=Tanh\left(W_{P}E_{I}\right)$$

(21) $$G=Sigmoid(W_{G}E_{I})$$

where $W_{P},W_{G}$ are learnable weights of a linear layer. The gating parameters are multiplied by the normalized weights and summed to yield the time interval weight β.

(22) $$\beta =G⊙E_{I}^{*}$$

Ultimately, the two weights are summed to compute the global state of the mission sequence.

(23) $$E_{G}=\left(\alpha +\beta \right)\cdot E_{M}$$

The collection of states for Sat is represented as $E_{GS}\in R^{N_{S}\times d}$.

### 4.2. Dynamic Request Modeling

Due to the uncertain number of dynamic requests, directly concatenating all information would increase the number of trainable parameters. Inspired by [64], we use a scalable mechanism to embed incoming requests. The structure of the embedding method is shown in Figure 2b.

Input requests $Req=m_{1},m_{2},\ldots ,m_{N_{R}}$ are first processed through a linear layer to obtain high-dimensional representations, followed by ReLU activation and another linear layer to generate request embeddings. For each $m_{i}$,

(24) $$e_{m_{i}}=W_{R_{2}}Relu\left(W_{R_{1}}m_{i}\right)$$

where $W_{R_{1}},W_{R_{2}}$ are learnable weights of linear layer. The vectors of all requests are denoted as $E_{m}$.

A pooling module is then used to generate the global state of the requests. Each request vector $e_{m_{i}}$ is passed through a linear layer with Tanh activation, followed by a Sigmoid-activated output layer to compute request weights.

(25) $$a_{m_{i}}=Sigmoid\left(W_{R_{4}}Tanh\left(W_{R_{3}}e_{m_{i}}\right)\right)$$

where $W_{R_{3}},W_{R_{4}}$ are learnable. We compute the weighted average of $E_{m}$ as the global state $E_{R}$ of the input requests.

(26) $$E_{R}=\sum_{i=1}^{N_{R}}a_{m_{i}}e_{m_{i}}$$

### 4.3. Multi-Satellite Mission Allocation

In our framework, mission allocation is modeled as a sequential decision problem, formulated as a Markov Decision Process (MDP) to apply DRL algorithms.

**Figure 4:**
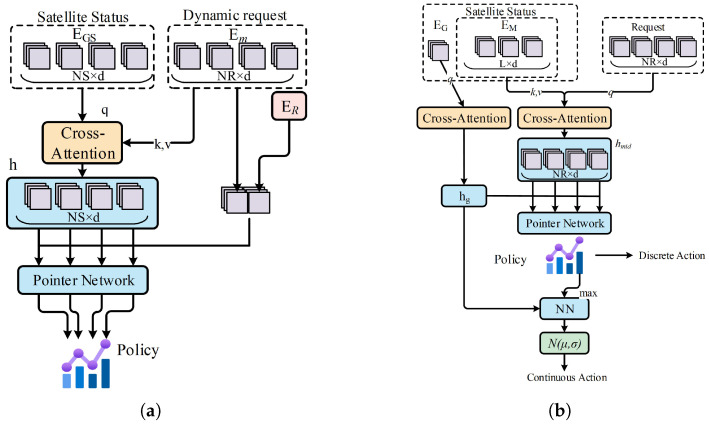
The actor network of MSRP. (**a**) Mission allocation. (**b**) Mission replanning.

1. State: In mission allocation, the state at step *t* comprises two parts: the mission sequence of satellites from t−1 and the dynamic request set. The satellites’ immediate state and resource changes are recorded within schedule. Using the mission sequence modeling method, we derive satellites’ global embedding $E_{GS}\in R^{N_{S}\times d}$. For dynamic requests, we generate the global embedding $E_{R}$ and individual request vectors as $e_{m_{i}}\in E_{m}$.
2. Action: The action space consists of satellites awaiting task allocation, defined as a discrete set. We employ a pointer mechanism to generate the policy function, with the detailed procedure outlined in the following steps and visually represented in Figure 4a.
  (a) Apply a cross-attention layer, where the query is the satellite state $E_{GS}\in R^{N_{S}\times d}$, and the key and value are the requests $E_{m}\in R^{N_{R}\times d}$. The output is the mission-satellite association vector $\left\{h_{1},h_{2},\ldots ,h_{N_{S}}\right\}$, which aims to capture the relationships between the satellite and all requests.
  (b) Concatenating each request vector $e_{m_{i}}$ with global embedding $E_{R}$ to construct vector $z=(e_{m_{i}},E_{R})$.
  (c) We use a pointer mechanism to generate the probability distribution of the allocation policy $\pi (a_{t}|s_{t})$.
    (27) $$u_{j}=v_{1}^{⊤}tanh\left(W_{P_{1}}h_{j}+W_{P_{2}}z\right)$$
    (28) $$\pi \left(a_{t}|s_{t}\right)=Softmax\left(u_{j}\right),j=1,2,\ldots ,N_{S}$$
3. Reward: The objective of mission allocation is to maximize the overall reward, which is determined after replanning by each satellite. Therefore, mission allocation is jointly optimized with the single-satellite replanning algorithm. If a request is successfully inserted into the plan, the step reward is the task’s revenue; otherwise, it returns zero. The revenue of each request is normalized by the sum of total revenues.

### 4.4. Single-Satellite Mission Replanning

To meet the mission replanning requirements in dynamic environments, a sequential decision-making algorithm for uncertain request arrivals has been developed. The objective is to maximize the task revenues while minimizing the changes to the original schedule. Single-satellite mission replanning can also be modeled as a Markov Decision Process.

1. State: In this replanning phase, the satellite state is represented by its mission sequence embedding $E_{M}\in R^{L\times d}$, which incorporates resource consumption. The global state $E_{G}\in R^{1\times d}$ is given by the pooling method. The dynamically arriving requests, with an uncertain scale $N_{R_{j}}$, are encoded and represented as $E_{m}\in R^{N_{R_{j}}\times d}$.
2. Action: The replanning actions are composite, using a hybrid action space. Each action at step *t* is represented as $a_{t}=\left(m_{i},Ts_{i}\right)$ where $m_{i}$ denotes the task selected from the allocated set, and $Ts_{i}$ refers to the execution time of the selected task. We introduce a termination action to end the replanning process. The action generation is handled by two branches of the actor network, which output discrete and continuous actions, respectively. The specific actor network is illustrated in Figure 4b.
  (a) State Extraction: Two consecutive cross-attention blocks are used to build a compressed representation of the current state. In the first block, $q=E_{m};k,v=E_{M}$, which generates the task-satellite affinity vector $h_{mid}\in R^{N_{R_{j}}\times d}$. In the second block, $q=E_{G};k,v=h_{mid}$, where the satellite’s global state is used as the query to capture the current global state $h_{g}$.
  (b) Discrete Action: The mission selection policy distribution $\pi_{d}\left(m_{t}|s_{t}\right)$ is generated using the pointer mechanism.
    (29) $$u_{i}=v_{2}^{⊤}tanh\left(W_{P_{3}}h_{mid}+W_{P_{4}}h_{g}\right)$$
    (30) $$\pi_{d}\left(m_{t}|s_{t}\right)=Softmax\left(u_{i}\right),i=1,2,\ldots ,N_{R_{j}}$$
  (c) Continuous Action: Add the selected task information into the actor network to generate the mean μ, and variance σ. After sampling from N(μ,σ), we scale the action to the range (−1,1) using the Tanh function. The output is then re-normalized to the specific mission’s start time using the formula.
    (31) $$\begin{array}{c} Ts_{i}=y_{c}*scale_{c}+bias_{c},\\ scale_{c}=\frac{s_{i}+e_{i}-d_{i}}{2},bias_{c}=\frac{e_{i}-s_{i}-d_{i}}{2} \end{array}$$
  (d) Mission Insertion: The mission $m_{i}$ is inserted based on $Ts_{i}$. In case of conflicts with existing missions, a fast insertion approach (FIA) like principle [19] is applied to resolve the conflict by shifting existing task or removing it if necessary.
3. Reward: Our optimization objective consists of two parts: mission revenues and the cost function associated with changes to the initial mission sequence. Thus, we design the reward as follows:
  (32) $$\begin{array}{c} r_{t}=\alpha \cdot \frac{len(t)}{len(t-1)+1}\cdot p_{sel}-\beta \cdot \sum p_{i}\cdot \tau_{change} \end{array}$$
  (33) $$\tau_{change}=\left\{\begin{array}{cc} -0.8, & \;if\;removed,\\ \frac{Ts_{i}\left(t\right)-Ts_{i}\left(t-1\right)}{e_{i}-s_{i}}, & \;if\;shifted,\\ 0, & \;if\;unchanged, \end{array}\right.$$
  where α,β are adjustable parameters that satisfy 0<α,β<1. len(t) represents the length of the satellite mission sequence at step *t*, $p_{i}$ denotes the revenue of $m_{i}$, and $\tau_{change}$ is the penalty factor for different levels of modifications. The goal of our reward design is to maximize both the task plan length and revenues while minimizing the disruption to the original plan during urgent request scheduling.

### 4.5. Training

We use proximal policy optimization (PPO) to sequentially train mission replanning and allocation policy. PPO is a policy-based reinforcement learning algorithm that estimates the performance of the policy using a novel objective with clipped probability ratios. The clipping function limits the magnitude of policy updates, ensuring stability and reliability during training.

We implement the DRL algorithm using the actor–critic architecture. The actor network outputs the policy $\pi (a_{t}|s_{t},\theta )$, while a critic network $V_{\phi }\left(s\right)$ is used as an estimator of the state-value function $V_{\pi }\left(s\right)$. The training process is illustrated as Algorithm 2.
**Algorithm 2** Procedure for training policy based on PPO  1:**Input:** Initial policy parameters $\theta_{0}$, initial value function parameters $\phi_{0}$, clipping parameter ϵ, discount factor γ, number of updates $N_{\mathrm{updates}}$, number of steps $N_{\mathrm{steps}}$, number of epochs *K*, learning rate α.  2:**Output:** Optimized policy parameters $\theta^{*}$  3:Initialize $\theta \leftarrow \theta_{0}$, $\phi \leftarrow \phi_{0}$. Initialize buffer *B*  4:**for** update $=1\to N_{\mathrm{updates}}$ **do**  5:   envs.reset()  6:   **for** step $=1\to N_{\mathrm{steps}}$ **do**  7:     Mission Sequence Modeling and Dynamic Request Modeling $s_{t}$  8:     Using current policy to generate $a_{t}∼\pi_{\theta }\left(a_{t}|s_{t}\right)$  9:     /*Do mission allocation or replanning*/10:     $s_{t+1},r_{t}\leftarrow \mathrm{envs}.\mathrm{step}\left(a_{t}\right)$11:     Store $\left\{s_{t},a_{t},r_{t}\right\}$ to buffer *B*12:   **end for**13:   **for** epoch =1→K **do**14:     Compute advantages using Generalized Advantage Estimation (GAE):15:     ${\hat{A}}_{t}=\sum_{l=0}^{T-t}{\left(\gamma \lambda \right)}^{l}\delta_{t+l}$, where $\delta_{t}=r_{t}+\gamma V_{\phi }\left(s_{t+1}\right)-V_{\phi }\left(s_{t}\right)$16:     Compute returns ${\hat{R}}_{t}={\hat{A}}_{t}+V_{\phi }\left(s_{t}\right)$17:     Update policy, value function using PPO objective:18:     $\theta \leftarrow \theta +\alpha \nabla_{\theta }E_{t}\left[\min\left(r_{t}\left(\theta \right){\hat{A}}_{t},\mathrm{clip}\left(r_{t}\left(\theta \right),1-\epsilon ,1+\epsilon \right){\hat{A}}_{t}\right)\right]$19:     $\phi \leftarrow \phi -\alpha \nabla_{\phi }E_{t}\left[{\left(V_{\phi }\left(s_{t}\right)-{\hat{R}}_{t}\right)}^{2}\right]$20:   **end for**21:**end for**22:**Return** Optimized policy parameters $\theta^{*}\leftarrow \theta$

The training process for both mission allocation and replanning agents is similar. The main difference is that mission allocation involves assigning requests to satellites sequentially, with the reward coming from the result of each satellite replanning. The general training flow is to first train the single-satellite replanning network and then fix the optimal replanning policy and integrate it into the mission allocation environment.

## 5. Computational Experiments

This section outlines the experimental setup and presents three simulations to validate our algorithm. Experiment 1 tests single-satellite replanning, Experiment 2 evaluates the mission allocation and replanning framework, and Experiment 3 conducts ablation studies to verify the method’s effectiveness.

### 5.1. Scenario Settings

The simulation scenario is based on the multi-satellite observation requirements and constellation parameters derived from the Walker Delta configuration. The constellation was created on 1 April 2020, with six orbital planes, each containing one satellite. The simulation time spans from 00:00:00 to 24:00:00 (UTC), aligning with an orbital period of 6080 seconds. Table 1 shows the specific orbital parameters of the satellites and Table 2 shows the setting of simulated observation requests.

The observation missions are generated as real-world application scenarios, distributed randomly within the geographic region from $3^{\circ }$ N to $53^{\circ }$ N and from $74^{\circ }$ E to $133^{\circ }$ E. The initial mission plan for each satellite consists of 100, 200, or 300 missions. The proportion of new dynamic requests per satellite relative to the initial plan size is set to 10%, 20%, 30%. In mission allocation, the number of requests is equal to the single-satellite capacity multiplied by the satellite count.

Training Setup. Based on the ADSMP algorithm from [65], we generate the optimal mission plans for a multi-satellite system. During training, we randomly select a certain proportion from the original plan as requests, with the remaining used as InitPlan. For validation, once InitPlan is determined, new observation requests are randomly generated each time.

The algorithm was implemented in Python 3.9.16, and the experiments were conducted on a system with an Intel(R) Xeon(R) 8375C 2.90 GHz processor, running Ubuntu 20.04, and equipped with a single NVIDIA GeForce RTX 4090 GPU.

For the single-satellite mission replanning algorithm, training is performed with 100 task size and 20% dynamic request ratio. For the mission allocation algorithm, the number of satellites is set to 4, with 100 task size and 20% dynamic request ratio, resulting in a total of 80 requests for training. The network parameters are shown in Table 3.

**Figure 5:**
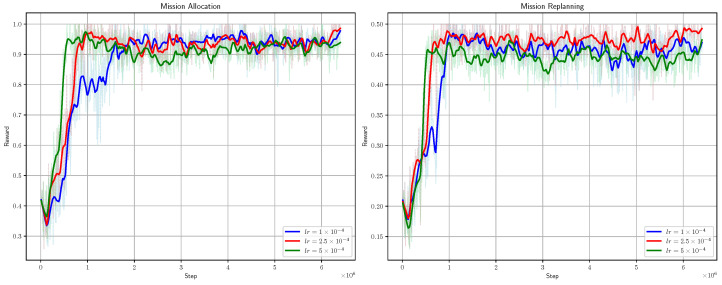
Training curves of MSRP allocation and replanning.

The convergence curve is shown in Figure 5. The fluctuations during the training process are mainly due to the randomness of the scenario’s input mission sequences.

The metrics considered in the experiment include:

1. Insert rate of new requests: $NIR=\frac{N_{ins}}{N_{R}}$;
2. Revenue rate of new request: $NRR=\frac{\sum_{i}^{N_{ins}}p_{i}}{\sum_{k}^{N_{R}}p_{k}}$;
3. Execution rate of missions: $ER=\frac{len(NewPlan)}{N_{R}+len\left(InitPlan\right)}$;
4. Total Revenue rate: $RR=\frac{\sum_{i}^{NewPlan}p_{i}}{\sum_{j}^{N_{R}}p_{j}+\sum_{k}^{InitPlan}p_{k}}$;
5. Modification rate: $MR=\frac{\sum_{i}^{InitPlan}p_{i}\cdot \tau_{change}}{\sum_{i}^{InitPlan}p_{i}}$;
6. Computation time: Time(s).

It is worth clarifying that MR is a metric to quantify the level of disturbance to the original plan. Based on Equations (3) and (33), we set the weight $\omega_{1}$ for removed tasks to −1, since these tasks are completely removed. The degree of shift is defined by the proportion of the change in execution time relative to the visible time window, which gives the weight $\omega_{2}$. We define MR as the ratio of the revenues before and after replanning.

### 5.2. Comparison Algorithms

We selected the following algorithms for comparison:

1. Multiple Strategies Local Replanning (MSLR) Algorithm: This algorithm integrates multiple insertion principles from [66]: direct insertion, move insertion, replace insertion; along with a hybrid insertion strategy from [7]: direct insertion, iterative insertion, conflict replacement insertion. Since our scenario focuses on mission-satellite visible time windows, we combined the merging insertion into direct insertion.
2. Transformer-based single-satellite replanning (TSR) Algorithm: We adopt the Transformer-based architecture with temporal encoding from [62] as the task scheduling method for single-satellite replanning. After obtaining discrete decisions, we use a neural network with the same architecture as MSRP to compute continuous actions.
3. Fast Insertion Approach (FIA) Algorithm: Based on the FIA principles from [19], this approach prioritizes tasks and determines insertion positions based on their feasibility between adjacent tasks in the satellite sequence, optimizing overall gain.
4. Multi-Satellite Replanning with adaptive Differential Evolution (RDE) Algorithm: We implemented an adaptive differential evolution algorithm [7] combined with the MSLR local replanning rule. The fitness function is defined as (1) with μ=0.5.
5. Transformer-based multi-satellite replanning (TMR) Algorithm: We combine the multi-satellite task allocation framework based on the attention mechanism from [67] with TSR as the single-satellite replanning method, forming our comparative algorithm.
6. Plan Regenerate (PRG) method: Based on the ADSMP method, we regenerate the task sequence by inputting both dynamic requests and the original missions.
7. Ablation-MLP: To validate the effectiveness of our mission sequence modeling module, we developed a baseline embedding network for comparison, represented by a three-layer MLP with 128 nodes. This network processes satellite mission sequence information and matches the input for the subsequent policy network. We used the PPO algorithm for training.
8. Ablation-pooling: To verify the effectiveness of our global pooling module, we replaced it with a mean pooling method for ablation. The PPO algorithm was used to train this version.
9. Ablation-Transformer-PE: Replace mission sequence modeling method by standard attention with classic position encoding (PE) for ablation.
10. Ablation-Transformer-RoPE: Replace mission sequence modeling method by standard attention with Rotary Position Embedding (RoPE) for ablation.

### 5.3. Experiment 1 on Single-Satellite Replanning

**Figure 6:**
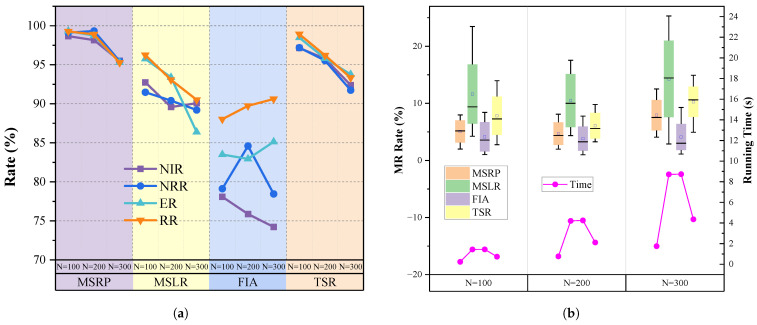
Experiment results with different single-satellite replanning algorithms under different numbers of missions. (**a**) Comparison of 4 metrics. (**b**) Comparison of Modification rate and running time.

**Figure 7:**
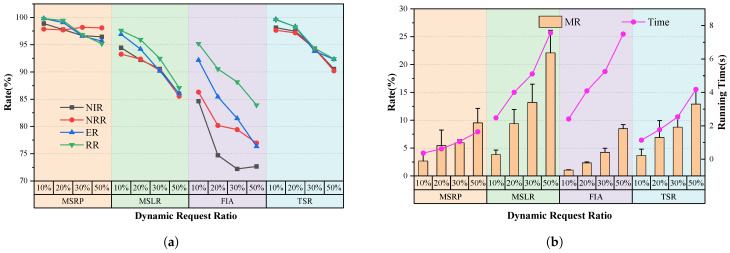
Experiment results with different single-satellite replanning algorithms under different new request ratio. (**a**) Comparison of of 4 metrics. (**b**) Comparison of Modification rate and running time.

We compared the replanning component of the MSRP algorithm with the MSLR algorithm, FIA algorithm and TSR algorithm. Figure 6 and Figure 7 present the performance evaluation of the four algorithms on varying scales of missions and request arrivals for single-satellite mission replanning.

When the scenario scale is relatively small, particularly 100 missions or 10% new requests, all algorithms respond quickly and achieve replanning with a low modification rate. The main reason is that smaller tasks and request scales leave ample idle time in the mission sequence, allowing for direct insertion. As task volume increases, all algorithms show a gradual decline in insertion rates and total revenue. However, our proposed algorithm consistently outperforms others across all scenarios. When the number of tasks reaches 300 or a new ratio of 50%, the average NIR and RR of MSRP remain around 95%, which is significantly higher than the other algorithms.

As more tasks are inserted, FIA’s reliance on simple interval assessments with a fixed 0.5 threshold for execution timing becomes ineffective, lowering the new task insertion rate. While FIA’s modification rate remains low without a replacement strategy, its limited insertion capacity leads to reduced overall revenue as requests increase.

The MSLR method uses complex insertion rules to handle overlapping conflicts that FIA cannot address. However, MSLR has the longest computation time due to iterative rule matching. Its limited flexibility in task insertion leads to frequent replacements, reducing overall revenues and increasing modifications to the original scheme, which can be proved in Figure 6b.

Compared to the TSR method, MSRP demonstrates significant performance improvements when handling 100 to 300 tasks. When the scenario scale is small, TSR and our model demonstrate similar performance, with metrics such as NIR, NRR, ER, and RR exceeding 95%. However, when the tasks increases to 300 or the proportion of new requests rises to 50%, MSPR maintains its metrics above 95%, while TSR’s performance drops significantly, with NRR and other metrics falling to around 90%. The downward trend suggests that as the number of tasks and the proportion of new requests increase, the performance gap between TSR and MSPR is likely to widen further, as illustrated in Figure 6a and Figure 7a.

We believe that the cumulative effect of replanning intensifies with each task insertion. As the proportion of new requests increases, the performance disparity between TSR and MSRP becomes more pronounced, primarily due to our algorithm’s enhanced precision in determining insertion positions with successive insertions. Compared to MSRP, TSR does not incorporate attitude information and can only perceive relative time differences between tasks, failing to address the effects of time required for attitude adjustments between tasks. As a result, it cannot effectively reduce the search space for execution times within the feasible insertion window, ultimately limiting its ability to improve replanning performance. Furthermore, our task sequence modeling approach introduces information about task intervals and durations through a global pooling module, effectively guiding new requests to be inserted into idle time slots of the sequence. This is evidenced by our method’s lower MR compared to TSR. As mentioned earlier, the pointer network has lower computational complexity than the Transformer, resulting in significantly reduced time consumption for MSRP. In scenarios with 300 tasks, the time required by MSRP is only 50% of that needed by TSR.

Our method leverages mission sequence modeling to directly determine the insertion approach. It effectively considers the relationships between the satellite state, the original plan, and incoming requests, providing an optimized replanning solution. In addition, it reduces computation time, enabling faster response.

**Figure 8:**
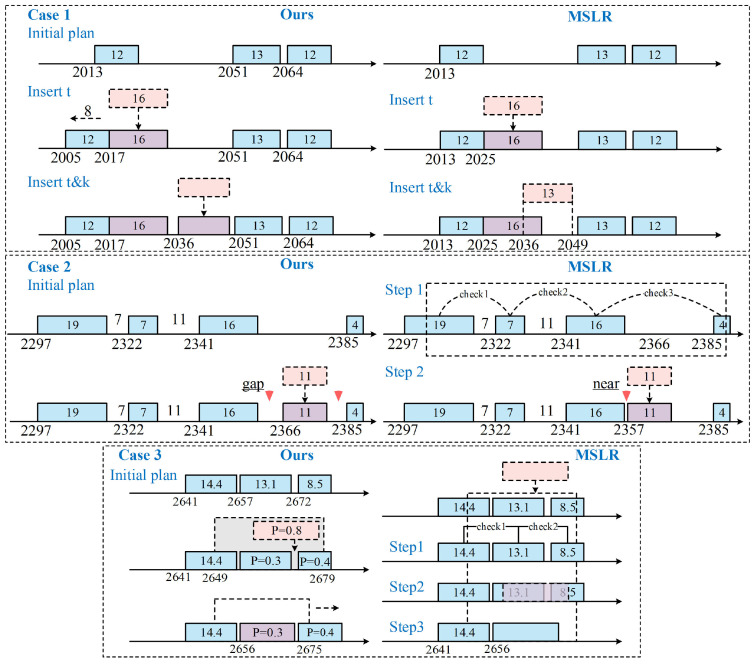
Three mission insertion cases.

The performance of our algorithm and MSLR is illustrated through three insertion cases in Figure 8. The number in the task block indicates its duration or priority. The dashed box marks the visible time window. Scheduled tasks are aligned on the timeline, with the numbers above indicating their start times.

Case 1 demonstrates direct task insertion. Our replanning algorithm assigns a specific start time for each task. $m_{t}$ is scheduled at 2017 s with a preceding task shifted forward. This creates a gap for subsequent tasks like $m_{k}$ to be inserted directly, which MSLR cannot achieve.

Case 2 shows the task insertion process. We directly insert $\left(m_{i},Ts_{i}\right)$, with gaps on both sides to allow future adjustments. In contrast, MSLR requires three rounds of checks to refuse Rule 1. Then, Rule 2 inserts the task after the preceding one, limiting flexibility for future adjustments. This case highlights that our method offers more flexibility and saves significant search time.

Case 3 shows a replacement insertion. MSLR searches three steps, confirms the new request’s reward (0.8) exceeds the combined reward of two original tasks (0.7) and proceeds with replacement. However, our method ensures the subsequent tasks meet the $T_{backward}$ constraint, enabling both replacement and shift insertion. This demonstrates that our algorithm achieves higher overall revenues in many cases.

Under different metrics, we conducted a “paired sample Wilcoxon signed-rank test” on the results of our model compared to other models, and calculated the confidence interval for the differences in results between the models. The results indicate that there is a significant difference between the outcomes of our model and those of the other models as shown in Table 4.

In summary, across experiments of varying scales, our proposed algorithm outperforms others in terms of insertion rate and revenue rate, while minimizing modifications to the original plans. As a result, it provides higher quality single-satellite replanning solutions.

### 5.4. Experiment 2 on Multi-Satellite Replanning Framework

**Figure 9:**
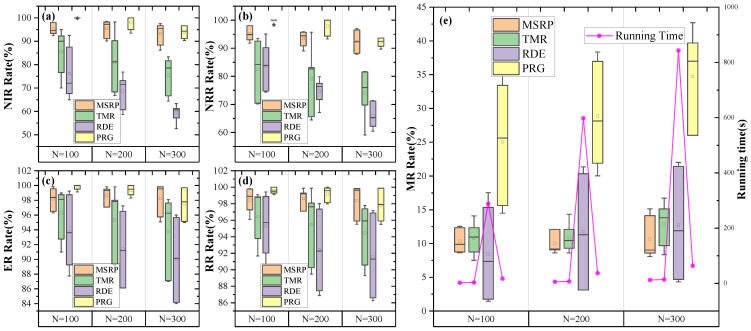
Experiment results with multi-satellite replanning algorithms under various number of missions. Subfigures (**a**–**d**) respectively present the comparison of NIR, NRR, ER, and RR results among the four algorithms under varying task quantities. Subfigure (**e**) displays the MR performance and running time of the different algorithms.

In this section, we validate the effectiveness of multi-satellite mission replanning across different scenarios. As shown in Figure 9, MSRP performs consistently well with 100–300 tasks, while other algorithms experience varying levels of decline, highlighting its strong replanning ability.

Our method outperforms RDE across various task volumes. Specifically, MSRP’s average NIR is 27.3% higher, and its NRR is 13.6% higher than RDE, demonstrating better performance in responding to dynamic requests. The efficient replanning ability of MSRP increases overall revenue, which is reflected in a high insertion rate accompanied by a high execution rate and a total revenue rate. As shown in Figure 9e, the MR of MSRP and RDE is similar at around 10%, but MSRP handles nearly 30% more new requests than RDE. This advantage stems from MSRP’s enhanced global state modeling and its ability to capture the relationship between new requests and the original mission sequence.

Compared to PRG, the results show that our replanning algorithm achieves a total revenue nearly identical to PRG, with only a 0.8% difference. However, as shown in Figure 9e, our MR is significantly lower than PRG, and its growth rate is slower as the scale increases. This demonstrates that our approach can achieve high-revenue task insertion while minimizing changes to the original sequence.

Compared to the learning-based TMR method, our approach outperforms it across task volumes ranging from 100 to 300, with less sensitivity to scale variations. Specifically, under the condition of consuming almost the same amount of time, NIR and NRR are 13.8% and 15.3% higher, while ER and RR are 3.2% and 3.1% higher, respectively, with a reduction of 1.2% in MR. By incorporating relative time differences and side swing angle differences into the replanning model, mission sequence modeling significantly enhances the model’s dynamic adaptability, enabling it to handle varying scales of input requests without being constrained by sequence order or frequent task insertions. Additionally, the proposed allocation method enhances the capability to perceive task insertion effects by capturing the correlation between satellite states and dynamic requests. Since our single-satellite replanning performance already exceeds TSR, combined with the flexibility of the pointer network in task allocation, our approach achieves more efficient single-satellite replanning, significantly improving both new request benefits and overall task benefits.

Figure 9 shows that our algorithm is the fastest, with a slow increase in computation time as the task scale grows. In contrast, the RDE algorithm involves multiple evolution iterations and rule matching during task insertion, leading to high time consumption, with solving times reaching hundreds of seconds. While PRG also uses neural network inference to speed up computation, it must plan all tasks in the scenario, which increases computational load and leads to higher time consumption than our approach. The results of the significance difference analysis and confidence interval of this experiment are shown in Table 5, indicating that there are significant differences between our model and other models.

### 5.5. Experiment 3 for Ablation

**Figure 10:**
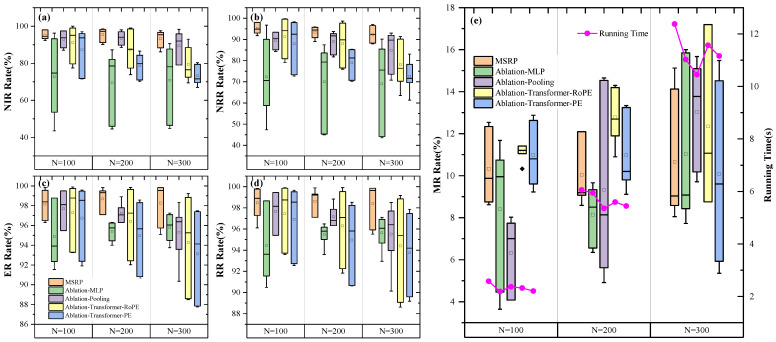
Ablation experiment results under different number of missions. Subfigures (**a**–**d**) respectively present the comparison of NIR, NRR, ER, and RR result under varying task quantities. Subfigure (**e**) displays the comparison of MR performance and running time.

The effectiveness of our method is validated through comparisons with ablation models. Results in Figure 10a show that MSRP outperforms Ablation-MLP by 23.3% in NIR and 17.2% in NRR. This highlights the importance of our proposed mission sequence modeling.

Mission sequence modeling also improves the model’s generalization. Trained in a 100–20% environment, MSRP shows stable performance across all test scenarios, with NIR and NRR standard deviations of 0.0401 and 0.088, respectively. In contrast, while ablation-MLP performs well in training-scale environments, its performance varies significantly across different scenarios, with standard deviations of 0.200 for NIR and 0.192 for NRR.

The comparison with the ablation-pooling confirms the effectiveness of the proposed gated global pooling mechanism. Results show that MSRP consistently outperforms across multiple metrics, especially as the task volume increases. We believe that the pooling mechanism uses mission time intervals to enhance the accuracy of the global state representation, facilitating better task allocation.

Compared to the standard Transformer with PE, MSRP improves NIR and NRR by 14.6% and 13.5%, respectively, and increases ER and RR by 3.5% each. Compared further to the ablation model with RoPE, MSRP achieves improvements of 8.9% and 7.5% in NIR and NRR, respectively, and 2.5% and 2.7% in ER and RR, with a 1.7% reduction in MR. Both methods use sequential numbering for positional information, limiting their effectiveness in replanning scenarios with frequent task insertion. In contrast to the standard attention model, RoPE calculates relative positional information, which we believe better preserves and enhances the modeling of relative differences between tasks. As a result, it outperforms the standard attention model using absolute positional information.

In our model, we replace the sequential numbering in rotational positional encoding with task execution times and side-swing angles. This relative positional encoding approach directly models the transition and maneuvering times between tasks, further enhancing the representation of mission sequences. Additionally, changes in sequence numbering during the replanning process do not affect this modeling. Ablation results demonstrate that our approach effectively supports replanning, improving task insertion rates and overall revenue while reducing MR. Table 6 presents a statistical analysis, showing significant differences between the results of this model and the ablated version.

### 5.6. Sensitivity Analysis

In this section, we analyze the sensitivity of several hyperparameters in MSRP.

Regarding Equation (33), α,β can be determined based on the real scheduling scenario. A larger α reflects a higher priority for urgent incoming requests, while a larger β prioritizes maintaining the original user requirements. We can adjust the weights through experimentation, and select the values that yield the optimal solution. In this paper, we set up imaging tasks through simulation, without a real mission scheduling context. Therefore, we assign both α,β as 0.5.

As $\omega_{1}$ and $\omega_{2}$ in the reward function, we believe that adjusting $\omega_{1}$ allows us to control the model’s mission insertion strategy and influence the conservativeness of task replacement. For $\omega_{2}$, we use the ratio of the changed time to VTW length as the weight, ensuring that $0<\omega_{2}<\omega_{1}$.

**Figure 11:**
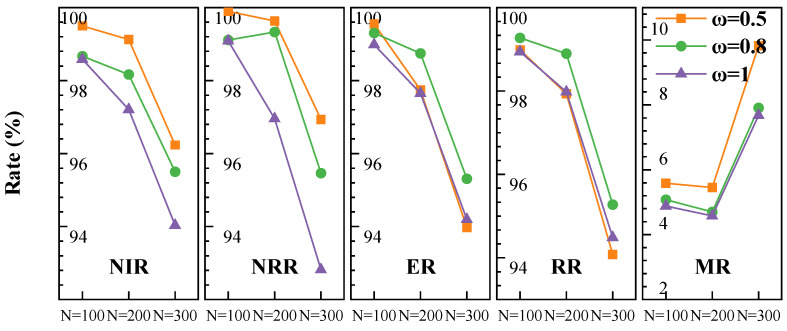
Sensitivity analysis of $\omega_{1}$ with different number of missions.

We conducted corresponding experiments by setting $\omega_{1}$ to 0.5, 0.8, and 1 to test the effects. The experimental results in Figure 11 show that when $\omega_{1}=0.5$, the NIR is relatively high, but the MR also increases, resulting in suboptimal overall task revenue. When set $\omega_{1}$ to 1, the insertion strategy is relatively conservative. However, overall revenue does not improve compared to $\omega_{1}=0.5$. We decided to set $\omega_{1}$ to 0.8, as it strikes a better balance between insertion rate and modification rate, leading to the highest overall revenue.

**Figure 12:**
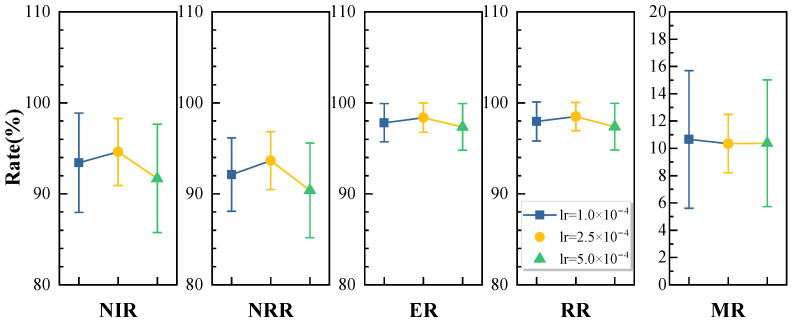
Sensitivity analysis of learning rate.

We trained the model with PPO learning rates of 1 × 10^−4^, 2.5 × 10^−4^, and 5 × 10^−4^, with the training curves shown in Figure 5. The convergence speed is slower with a smaller learning rate, and faster with a larger one. As seen in Figure 12, the models with learning rates of 1 × 10^−4^ and 2.5 × 10^−4^ perform similarly, while the model with lr = 5 × 10^−4^ shows a slight drop in performance as the task scale increases. The model with lr = 2.5 × 10^−4^ also has the smallest standard deviation across metrics in different scenarios, indicating better strategy adaptation. Therefore, we chose 2.5 × 10^−4^ as our optimal learning rate.

**Figure 13:**
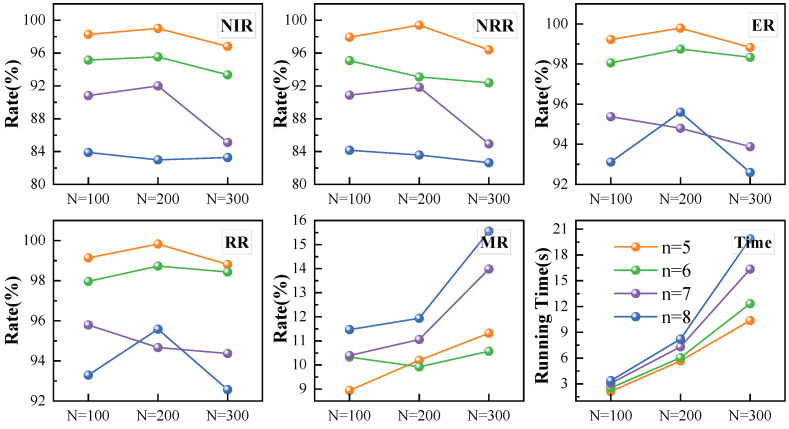
Sensitivity analysis of the number of satellite with different number of missions.

Figure 13 illustrates the performance of MSRP under different task volumes and satellite counts. Here, we use sat 1 as the seed satellite and use the Walker constellation architecture, with each satellite occupying a separate orbital plane to generate the test scenarios.

We generate dynamic requests as a fixed proportion of each satellite’s original task volume. As the number of satellites increases, the total number of requests also grows. As the satellite count rises from five to eight, the performance of replanning gradually declines. Specifically, NIR and NRR decrease notably, while ER and RR show smaller declines. This is due to the increased task volume with more satellites, reducing the available space for new insertion. Despite this, overall revenue remains around 92%, demonstrating the adaptability and effectiveness of our method across varying scales.

Furthermore, when the task volume per satellite is 100 or 200, our method shows stable performance across all metrics. When the volume reaches 300, there is a slight performance decline, with NRR and RR still maintaining above 82% and 92%, respectively. This indicates that our model performs well in larger-scale scenarios.

## 6. Conclusions

The multi-satellite observation mission replanning problem under dynamic arrival of requests is addressed in this work. Our goal is to maximize the revenues after replanning while minimizing changes to the original plan. Currently, no studies directly model the original task sequence. To address this, we introduce a mission sequence modeling method based on time-attitude rotational position encoding, which enhances the capture of correlations between tasks. Additionally, we design a global pooling module that incorporates interval information to generate a compact representation for satellite states. We propose a multi-satellite mission replanning framework comprising two components: mission allocation and replanning. For each module, we design neural network-based policy functions and optimize them using the PPO algorithm. Computational experiments demonstrate that our approach effectively generates solutions for arriving observation requests, outperforming state-of-the-art methods in terms of new request insertion revenue rate (15.27% improvement), overall revenue rate (3.05% increase) and modification rate (1.17% reduction), while achieving shorter computation times. Furthermore, ablation studies validate the effectiveness of the proposed techniques.

Although the decoder uses a pointer network, the encoder must recalculate the mission sequence modeling at each step based on the replanning scheme. This becomes computationally expensive as the sequence length *L* increases. Future work could explore incremental computation techniques to improve efficiency. Furthermore, our task allocation method depends on the quality of the single-satellite replanning strategy, which may limit the generalization of the task allocation policy. A possible improvement would be to consider joint optimization across the entire system. Lastly, the hybrid action space used to generate replanning strategies requires careful design of the reward function. Future research on multi-satellite observation mission replanning may be oriented in two key directions. While our study focuses on fast mission allocation by individual satellites, simultaneous allocation by multiple satellites will require effective conflict resolution strategies when the satellite number increases. In addition, incorporating environmental factors such as cloud cover and light conditions into the replanning process could improve image quality and mission benefits, making it a valuable direction for further exploration.

## Figures and Tables

**Table 1:** The orbital parameters of 6 satellites.

Satellite	*a* (km)	*e*	*I* (°)	ω (°)	Ω (°)	*M* (°)
Sat1	7200.0	0.000627	96.5760	0	175.72	0.0750
Sat2	7200.0	0.000627	96.5760	0	145.72	30.0750
Sat3	7200.0	0.000627	96.5760	0	115.72	60.0750
Sat4	7200.0	0.000627	96.5760	0	85.72	90.0750
Sat5	7200.0	0.000627	96.5760	0	55.72	120.0750
Sat6	7200.0	0.000627	96.5760	0	25.72	150.0750

**Table 2:** The parameters of simulated observation requests.

Parameters	Value	Unit	Description
$d_{i}^{j}$	[5,25)	s	Required execution duration
$p_{i}$	[0.1, 0.9]	1	Revenue (priority) of request
$a_{i}^{j}$	[−50, 50]	1	Side swing angle range
$C_{mem}^{j}$	1000	GB	Memory capacity
${mem}_{i}$	$1\times d_{i}^{j}$	GB	Memory consumption
$C_{power}^{j}$	512	W· h	Power capacity
${power}_{i}$	$\left[0.3,0.8\right)\times d_{i}^{j}$	W· h	Power consumption
$v^{j}$	1	s/deg	Time per degree of transition

**Table 3:** The hyperparameters of algorithms.

Hyperparameters	Replanning	Allocation
Number of envs	128	32
Total timesteps	64 × 10^5^	64 × 10^5^
Mini-batches	32	8
Learning rate	2.5 × 10^−4^	2.5 × 10^−4^
Learning rate decay	CosineAnnealing	StepAnnealing
γ	0.99	0.99
GAE λ	0.95	0.95
Update epochs	8	4
Cliping coefficient	0.2	0.1
Entropy coefficiant	0.01	0.01
Embedding dimension	128	128
Gate embedding dimension	32	32

**Table 4:** Statistical significance and confidence intervals for single-satellite replanning algorithm performance.

Algorithm Comparison	Statistical Indicators	NIR	NRR	ER	RR	MR	Time	Significant Difference (y/n)
MSRP|MSLR	Wilcoxon signed ranks test *p*-value	0.0033	0.0033	0.0033	0.0033	0.001	0.0005	y
	Confidence Interval (X1–X2)	[0.0425, 0.0902]	[0.0489, 0.1031]	[0.0359, 0.0830]	[0.0265, 0.0642]	[−0.0934, −0.0312]	[−5.7909, −1.9408]	
MSRP|FIA	Wilcoxon signed ranks test *p*-value	0.0005	0.0005	0.0005	0.0005	0.0015	0.0005	y
	Confidence Interval (X1–X2)	[0.1791, 0.2486]	[0.1392, 0.2057]	[0.1017, 0.1769]	[0.0557, 0.1114]	[0.0076, 0.0298]	[−5.8088, −1.9696]	
MSRP|TSR	Wilcoxon signed ranks test *p*-value	0.0077	0.0077	0.0076	0.0077	0.0005	0.0005	y
	Confidence Interval (X1–X2)	[0.0077, 0.0390]	[0.0090, 0.0538]	[0.0032, 0.0321]	[0.0032, 0.0299]	[−0.0316, −0.0116]	[−2.2554, −0.7101]	

**Table 5:** Statistical significance and confidence intervals for multi-satellite replanning algorithm performance.

Algorithm Comparison	Statistical Indicators	NIR	NRR	ER	RR	MR	Time	Significant Difference (y/n)
MSRP|TMR	Wilcoxon signed ranks test *p*-value	0.0004	0.0003	0.0004	0.0003	0.0076	0	y
	Confidence Interval (X1–X2)	[0.0986, 0.1787]	[0.1111, 0.1915]	[0.0177, 0.0474]	[0.0174, 0.0412]	[−0.0200, −0.0035]	[−1.3985, −0.7713]	
MSRP|RDE	Wilcoxon signed ranks test *p*-value	0	0	0.0004	0.0003	0.6397	0	y
	Confidence Interval (X1–X2)	[0.2239, 0.3071]	[0.1527, 0.2239]	[0.0177, 0.0474]	[0.0174, 0.0412]	[−0.0339, 0.0213]	[−686.6162, −452.3476]	
MSRP|PRG	Wilcoxon signed ranks test *p*-value	0.0023	0.0013	0	0.0394	0	0	y
	Confidence Interval (X1–X2)	[−0.0407, −0.0134]	[−0.0444, −0.0145]	[0.0484, 0.0850]	[−0.0096, 0.0022]	[−0.2271, −0.1573]	[−40.1716, −24.7170]	

**Table 6:** Statistical significance and confidence intervals for ablation experiments.

Algorithm Comparison	Statistical Indicators	NIR	NRR	ER	RR	MR	Time	Significant Difference (y/n)
MSRP| Ablation-MLP	Wilcoxon signed ranks test *p*-value	0.0002	0.0001	0.0007	0.0002	0.1297	0.1084	y
	Confidence Interval (X1–X2)	[0.1291, 0.3426]	[0.1290, 0.3346]	[0.0170, 0.0433]	[0.0201, 0.0463]	[−0.0002, 0.0232]	[−0.0273, 1.2680]	
MSRP| Ablation-pooling	Wilcoxon signed ranks test *p*-value	0.0004	0	0	0	0.3927	0.0001	y
	Confidence Interval (X1–X2)	[0.0113, 0.0411]	[0.0355, 0.0817]	[0.0098, 0.0228]	[0.0103, 0.0241]	[−0.0096, 0.0252]	[0.3827, 1.5095]	
MSRP|Ablation- Transformer-RoPE	Wilcoxon signed ranks test *p*-value	0.0013	0.001	0.0023	0.0016	0.0001	0.0001	y
	Confidence Interval (X1–X2)	[0.0470, 0.1266]	[0.0375, 0.1196]	[0.0113, 0.0363]	[0.0121, 0.0371]	[−0.0242, −0.0105]	[0.2066, 0.8139]	
MSRP|Ablation- Transformer-PE	Wilcoxon signed ranks test *p*-value	0	0	0.0001	0.0001	0.0268	0	y
	Confidence Interval (X1–X2)	[0.1077, 0.1899]	[0.0976, 0.1791]	[0.0211, 0.0476]	[0.0208, 0.0450]	[−0.0092, 0.0024]	[0.4075, 1.0628]	

## Data Availability

The data presented in this study are all contained within this article.

## References

[1] Lemaître M., Verfaillie G., Jouhaud F., Lachiver J., Bataille N. (2002). Selecting and scheduling observations of agile satellites. Aerosp. Sci. Technol..

[2] Crisp N.H., Roberts P.C.E., Livadiotti S., Oiko V.T.A., Edmondson S., Haigh S.J., Huyton C., Sinpetru L.A., Smith K.L., Worrall S.D. (2020). The benefits of very low earth orbit for earth observation missions. Prog. Aerosp. Sci..

[3] Zheng Z. (2019). Autonomous Onboard Mission Planning for Multiple Satellite Systems. Ph.D. Thesis.

[4] Cui J., Zhang X. (2019). Application of a Multi-Satellite Dynamic Mission Scheduling Model Based on Mission Priority in Emergency Response. Sensors.

[5] Lu Z., Shen X., Li D., Cheng S., Wang J., Yao W. (2024). Super-agile satellites imaging mission planning method considering degradation of image MTF in dynamic imaging. Int. J. Appl. Earth Obs. Geoinf..

[6] Xiao Y., Zhang S., Yang P., You M., Huang J. (2019). A two-stage flow-shop scheme for the multi-satellite observation and data downlink scheduling problem considering weather uncertainties. Reliab. Eng. Syst. Saf..

[7] Yang X., Hu M., Huang G., Li A. (2023). A Hybrid Local Replanning Strategy for Multi-Satellite Imaging Mission Planning in Uncertain Environments. IEEE Access.

[8] Wang J., Zhu X., Qiu D., Yang L.T. (2014). Dynamic Scheduling for Emergency Tasks on Distributed Imaging Satellites with Task Merging. IEEE Trans. Parallel Distrib. Syst..

[9] Chong W., Jun L., Ning J., Jun W., Hao C. (2011). A Distributed Cooperative Dynamic Task Planning Algorithm for Multiple Satellites Based on Multi-agent Hybrid Learning. Chin. J. Aeronaut..

[10] Li H., Li Y., Liu Y., Zhang K., Li X., Li Y., Zhao S. (2024). A Multi-Objective Dynamic Mission-Scheduling Algorithm Considering Perturbations for Earth Observation Satellites. Aerospace.

[11] Yang Y., Liu D. (2023). Distributed Imaging Satellite Mission Planning Based on Multi-Agent. IEEE Access.

[12] Sun H., Xia W., Hu X., Xu C. (2019). Earth observation satellite scheduling for emergency tasks. J. Syst. Eng. Electron..

[13] He L., Liu X.L., Chen Y.W., Xing L.N., Liu K. (2019). Hierarchical scheduling for real-time agile satellite task scheduling in a dynamic environment. Adv. Space Res..

[14] Li H., Li Y., Meng Q.Q., Li X., Shao L., Zhao S. (2024). An onboard periodic rescheduling algorithm for satellite observation scheduling problem with common dynamic tasks. Adv. Space Res..

[15] Li K., Zhang T., Wang R. (2021). Deep Reinforcement Learning for Multiobjective Optimization. IEEE Trans. Cybern..

[16] Pemberton J.C., Greenwald L. (2002). On the need for dynamic scheduling of imaging satellites. Int. Arch. Photogramm. Remote Sens. Spat. Inf. Sci..

[17] Liang J., Zhu Y.H., Luo Y.Z., Zhang J.C., Zhu H. (2021). A precedence-rule-based heuristic for satellite onboard activity planning. Acta Astronaut..

[18] Wen J., Liu X., He L. (2021). Real-time online rescheduling for multiple agile satellites with emergent tasks. J. Syst. Eng. Electron..

[19] Han C., Gu Y., Wu G., Wang X. (2023). Simulated Annealing-Based Heuristic for Multiple Agile Satellites Scheduling Under Cloud Coverage Uncertainty. IEEE Trans. Syst. Man Cybern.-Syst..

[20] Liu S., Chen Y., Xing L., Guo X. (2016). Time-dependent autonomous task planning of agile imaging satellites. J. Intell. Fuzzy Syst..

[21] Wei L., Xing L., Wan Q., Song Y., Chen Y. (2021). A Multi-objective Memetic Approach for Time-dependent Agile Earth Observation Satellite Scheduling Problem. Comput. Ind. Eng..

[22] Peng G., Song G., He Y., Yu J., Xiang S., Xing L., Vansteenwegen P. (2022). Solving the Agile Earth Observation Satellite Scheduling Problem With Time-Dependent Transition Times. IEEE Trans. Syst. Man Cybern.-Syst..

[23] Du B., Li S. (2019). A new multi-satellite autonomous mission allocation and planning method. Acta Astronaut..

[24] Du Y., Wang T., Xin B., Wang L., Chen Y., Xing L. (2020). A Data-Driven Parallel Scheduling Approach for Multiple Agile Earth Observation Satellites. IEEE Trans. Evol. Comput..

[25] Song Y., Xing L., Wang M., Yi Y., Xiang W., Zhang Z. (2020). A knowledge-based evolutionary algorithm for relay satellite system mission scheduling problem. Comput. Ind. Eng..

[26] Zhu W., Hu X., Xia W., Jin P. (2019). A two-phase genetic annealing method for integrated Earth observation satellite scheduling problems. Soft Comput..

[27] Wang Y., Liu D., Liu J. (2024). A Bilevel Programming Model for Multi-Satellite Cooperative Observation Mission Planning. IEEE Access.

[28] Bianchessi N., Cordeau J.F., Desrosiers J., Laporte G., Raymond V. (2007). A heuristic for the multi-satellite, multi-orbit and multi-user management of Earth observation satellites. Eur. J. Oper. Res..

[29] Li J., Chen Y., Liu X., He R. JADE implemented multi-agent based platform for multiple autonomous satellite system. Proceedings of the 2018 SpaceOps Conference.

[30] Qi J., Guo J., Wang M., Wu C. (2021). A Cooperative Autonomous Scheduling Approach for Multiple Earth Observation Satellites With Intensive Missions. IEEE Access.

[31] Liu Y., Chen Q., Li C., Wang F. (2021). Mission planning for Earth observation satellite with competitive learning strategy. Aerosp. Sci. Technol..

[32] Chen Y., Tian G., Guo J., Huang J. (2021). Task Planning for Multiple-Satellite Space-Situational-Awareness Systems. Aerospace.

[33] Yang W., He L., Liu X., Chen Y. (2021). Onboard coordination and scheduling of multiple autonomous satellites in an uncertain environment. Adv. Space Res..

[34] Li J., Yang A., Jing N., Hu W. Coordinated planning of space-aeronautics earth-observing based on CSP theory. Proceedings of the 2013 21st International Conference on Geoinformatics.

[35] Liu D., Dou L., Zhang R., Zhang X., Zong Q. (2023). Multi-Agent Reinforcement Learning-Based Coordinated Dynamic Task Allocation for Heterogenous UAVs. IEEE Trans. Veh. Technol..

[36] Dalin L., Haijiao W., Zhen Y., Yanfeng G., Shi S. (2021). An Online Distributed Satellite Cooperative Observation Scheduling Algorithm Based on Multiagent Deep Reinforcement Learning. IEEE Geosci. Remote Sens. Lett..

[37] Saeed A.K., Holguin F., Yasin A.S., Johnson B.A., Rodriguez B.M. Multi-Agent and Multi-Target Reinforcement Learning for Satellite Sensor Tasking. Proceedings of the 2024 IEEE Aerospace Conference.

[38] Zhang G., Li X., Hu G., Li Y., Wang X., Zhang Z. (2023). MARL-Based Multi-Satellite Intelligent Task Planning Method. IEEE Access.

[39] Xhafa F., Ip A.W. (2021). Optimisation problems and resolution methods in satellite scheduling and space-craft operation: A survey. Enterp. Inf. Syst..

[40] Cho D.H., Kim J.H., Choi H.L., Ahn J. (2018). Optimization-Based Scheduling Method for Agile Earth-Observing Satellite Constellation. J. Aerosp. Inf. Syst..

[41] Kim J., Ahn J., Choi H.L., Cho D.H. (2020). Task Scheduling of Agile Satellites with Transition Time and Stereoscopic Imaging Constraints. J. Aerosp. Inf. Syst..

[42] Li P., Li J., Li H., Zhang S., Yang G. Graph Based Task Scheduling Algorithm for Earth Observation Satellites. Proceedings of the 2018 IEEE Global Communications Conference (GLOBECOM).

[43] Gabrel V., Moulet A., Murat C., Paschos V.T. (1997). A new single model and derived algorithms for the satellite shot planning problem using graph theory concepts. Ann. Oper. Res..

[44] Valicka C.G., Garcia D., Staid A., Watson J.P., Hackebeil G., Rathinam S., Ntaimo L. (2019). Mixed-integer programming models for optimal constellation scheduling given cloud cover uncertainty. Eur. J. Oper. Res..

[45] Song Y., Xing L., Chen Y. (2022). Two-stage hybrid planning method for multi-satellite joint observation planning problem considering task splitting. Comput. Ind. Eng..

[46] Chen Y., Lu J., He R., Ou J. (2020). An Efficient Local Search Heuristic for Earth Observation Satellite Integrated Scheduling. Appl. Sci..

[47] Luo Q., Peng W., Wu G., Xiao Y. (2022). Orbital Maneuver Optimization of Earth Observation Satellites Using an Adaptive Differential Evolution Algorithm. Remote Sens..

[48] Li Y., Luo J., Zhang W., Xiang F. (2024). Genetic-evolutionary bi-level mission planning algorithm for multi-satellite cooperative observation. Syst. Eng. Electron..

[49] Zheng Z., Guo J., Gill E. (2018). Onboard autonomous mission re-planning for multi-satellite system. Acta Astronaut..

[50] Mao H., Alizadeh M., Menache I., Kandula S. (2016). Resource Management with Deep Reinforcement Learning. Proceedings of the 15th ACM Workshop on Hot Topics in Networks, Atlanta GA USA.

[51] Chen H., Luo Z., Peng S., Wu J., Li J. (2022). HiPGen: An approach for fast generation of multi-satellite observation plans via a hierarchical multi-channel transformer network. Adv. Space Res..

[52] Gu Y., Han C., Chen Y., Xing W.W. (2022). Mission Replanning for Multiple Agile Earth Observation Satellites Based on Cloud Coverage Forecasting. IEEE J. Sel. Top. Appl. Earth Obs. Remote Sens..

[53] Wang H., Yang Z., Zhou W., Li D. (2019). Online scheduling of image satellites based on neural networks and deep reinforcement learning. Chin. J. Aeronaut..

[54] Schuetz M.J., Brubaker J.K., Katzgraber H.G. (2022). Combinatorial optimization with physics-inspired graph neural networks. Nat. Mach. Intell..

[55] Wang Z., Hu X., Ma H., Xia W. (2024). Learning multi-satellite scheduling policy with heterogeneous graph neural network. Adv. Space Res..

[56] Feng X., Li Y., Xu M. (2024). Multi-satellite cooperative scheduling method for large-scale tasks based on hybrid graph neural network and metaheuristic algorithm. Adv. Eng. Inform..

[57] Nazari M., Oroojlooy A., Takáč M., Snyder L.V. (2018). Reinforcement learning for solving the vehicle routing problem. Proceedings of the 32nd International Conference on Neural Information Processing Systems.

[58] Kool W., van Hoof H., Welling M. Attention, Learn to Solve Routing Problems! In Proceedings of the International Conference on Learning Representations, New Orleans, LA, USA, 6–9 May 2019.

[59] Liu Z., Xiong W., Han C., Yu X. (2024). Deep Reinforcement Learning with Local Attention for Single Agile Optical Satellite Scheduling Problem. Sensors.

[60] Chen M., Du Y., Tang K., Xing L., Chen Y., Chen Y. (2024). Learning to Construct a Solution for the Agile Satellite Scheduling Problem With Time-Dependent Transition Times. IEEE Trans. Syst. Man Cybern. Syst..

[61] Liang J., Liu J.P., Sun Q., Zhu Y.H., Zhang Y.C., Song J.G., He B.Y. (2023). A Fast Approach to Satellite Range Rescheduling Using Deep Reinforcement Learning. IEEE Trans. Aerosp. Electron. Syst..

[62] Long Y., Shan C., Shang W., Li J., Wang Y. (2024). Deep Reinforcement Learning-Based Approach With Varying-Scale Generalization for the Earth Observation Satellite Scheduling Problem Considering Resource Consumptions and Supplements. IEEE Trans. Aerosp. Electron. Syst..

[63] Jiang Y., Yang Y., Xu Y., Wang E. (2024). Spatial-Temporal Interval Aware Individual Future Trajectory Prediction. IEEE Trans. Knowl. Data Eng..

[64] Agrawal A., Bedi A.S., Manocha D. RTAW: An Attention Inspired Reinforcement Learning Method for Multi-Robot Task Allocation in Warehouse Environments. Proceedings of the 2023 IEEE International Conference on Robotics and Automation (ICRA).

[65] Li P., Wang H., Zhang Y., Pan R. (2024). Mission planning for distributed multiple agile Earth observing satellites by attention-based deep reinforcement learning method. Adv. Space Res..

[66] Sun H., Xia W., Wang Z., Hu X. (2021). Agile earth observation satellite scheduling algorithm for emergency tasks based on multiple strategies. J. Syst. Sci. Syst. Eng..

[67] Ou J., Xing L., Yao F., Li M., Lv J., He Y., Song Y., Wu J., Zhang G. (2023). Deep reinforcement learning method for satellite range scheduling problem. Swarm Evol. Comput..

